# Gene expression profiling data of *Schizosaccharomyces pombe* under nitrosative stress using differential display

**DOI:** 10.1016/j.dib.2015.11.047

**Published:** 2015-12-02

**Authors:** Pranjal Biswas, Uddalak Majumdar, Sanjay Ghosh

**Affiliations:** Department of Biochemistry, University of Calcutta, 35, Ballygunge Circular Road, Kolkata 700019, West Bengal, India

**Keywords:** *Schizosaccharomyces pombe*, Nitric oxide, Nitrosative stress, Differential display analysis, NO, Nitric oxide, RNIs, Reactive nitrogen intermediates, DAVID, Database for annotation, visualization and integrated discovery, GO, Gene ontology, KEGG, Kyoto encyclopedia of genes and genomes

## Abstract

Excess production of nitric oxide (NO) and reactive nitrogen intermediates (RNIs) causes nitrosative stress on cells. *Schizosaccharomyces pombe* was used as a model to study nitrosative stress response. In the present data article, we have used differential display to identify the differentially expressed genes in the fission yeast under nitrosative stress conditions. We have used pure NO donor compound detaNONOate at final concentrations of 0.1 mM and 1 mM to treat the cells for 15 min alongside control before studying their gene expression profiles. At both the treated conditions, we identified genes which were commonly repressed while several genes were induced upon both 0.1 mM and 1 mM treatments. The differentially expressed genes were further analyzed in DAVID and categorized into several different pathways.

**Specifications table**

**Table:** 

Subject area	*Biology*
More specific subject area	*Molecular biology*
Type of data	*Tables and figures*
How data was acquired	*Polymerase Chain Reaction (Applied Biosystems PCR System 9700), Denaturing Poly-acrylamide Gel Electrophoresis (Bio-Rad Sequi-Gen GT System)*
*DNA sequencing using BigDye® Terminator v3*.*1 Cycle Sequencing Kit (Applied Biosystems) and Applied Biosystems3700 sequencer.*
Data format	*Analyzed using statistical tests*
Experimental factors	*Fission yeast cells were treated with pure NO donor compound detaNONOate at final concentrations of 0.1* *mM and 1* *mM alongside controls for 15 min.*
Experimental features	*RNA was extracted from the control and treated cells and converted to cDNA. PCR was performed using degenerate primers, products separated by denaturing poly-acrylamide gel electrophoresis. Differentially expressed transcripts were sequenced to identify the genes.*
Data source location	*Department of Biochemistry, University of Calcutta, Kolkata, West Bengal, India*
Data accessibility	*Data is with this article*

**Value of the data**

• The data shows the differentially expressed genes in the fission yeast under nitrosative stress which could be compared to differentially expressed genes in other stress conditions
• Comparison of the data with differentially expressed genes in other stress conditions could help in better understanding of the gene expression patterns under nitrosative stress
• The affected pathways under nitrosative stress could be compared to those affected under other stress conditions
• Based on the data detailed pathway oriented studies could be undertaken to understand the mechanism of nitrosative stress action

## 1 Data

**Fig. 1:**
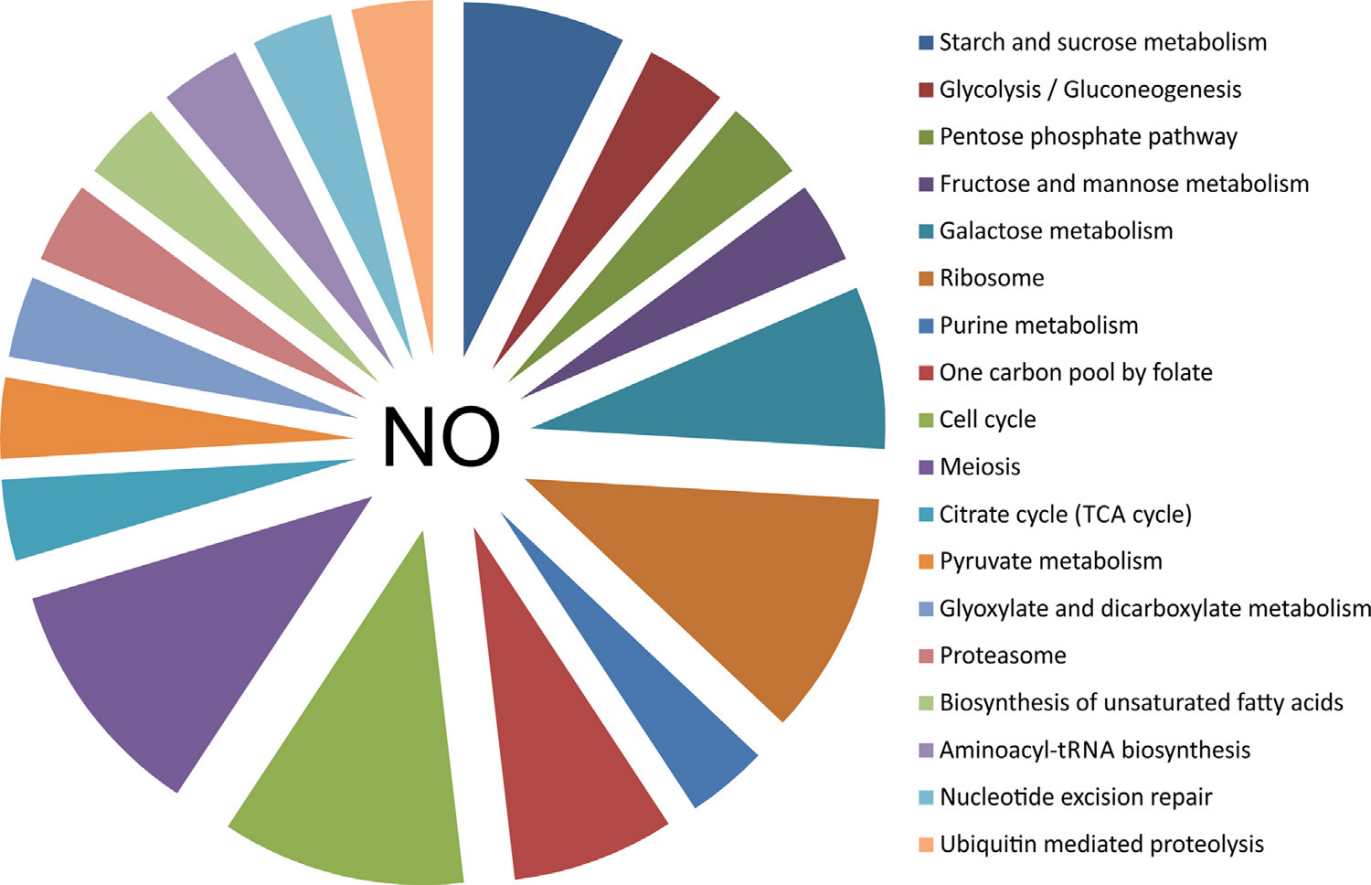
Pathways affected when fission yeast cells are treated with 0.1 mM NO donor compound. Pathway analysis has been performed from the list of differentially expressed genes using the online tool DAVID and as per information from KEGG.

**Fig. 2:**
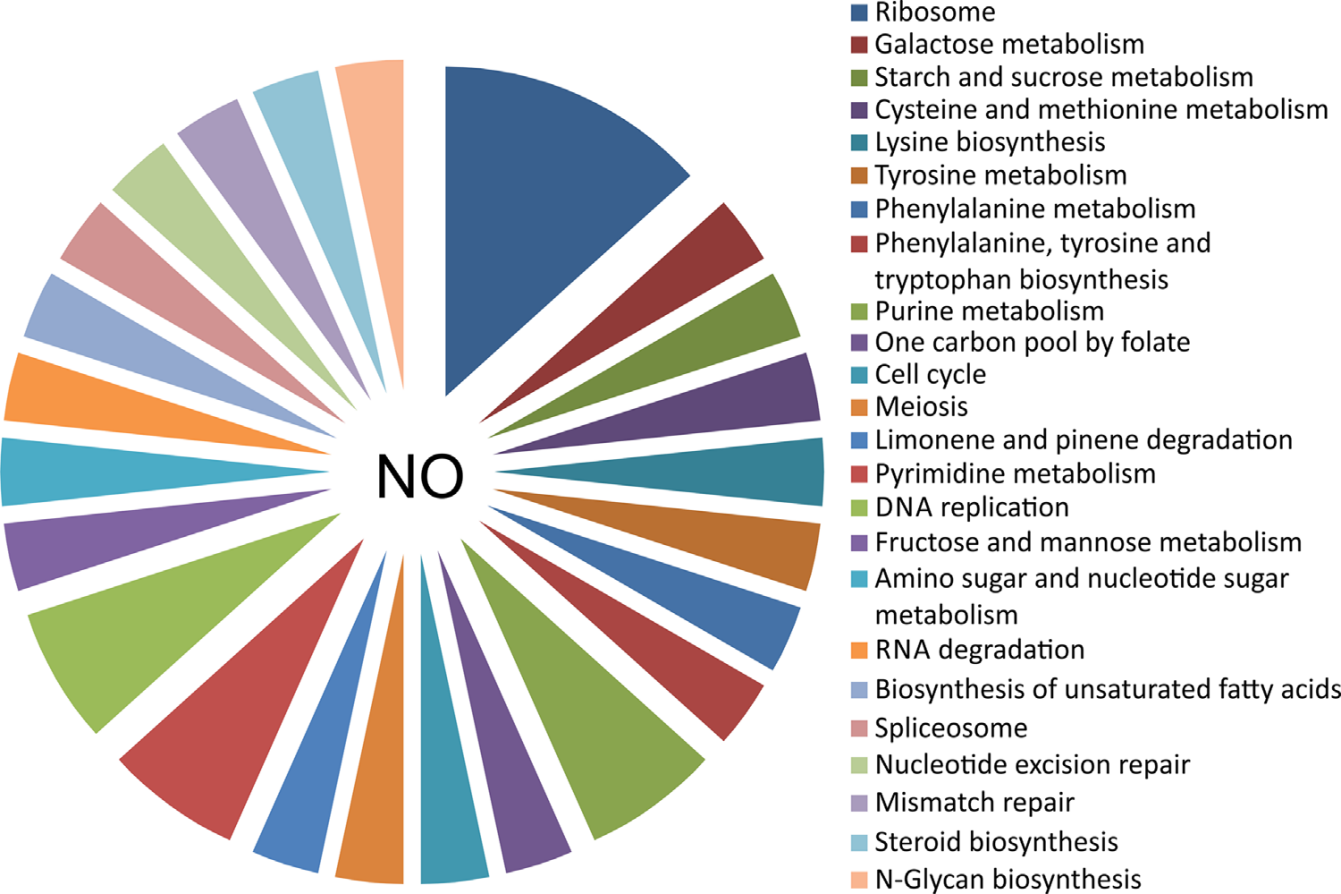
Pathways affected when fission yeast cells are treated with 1 mM NO donor compound. Pathway analysis has been performed from the list of differentially expressed genes using the online tool DAVID and as per information from KEGG.

To identify the differentially expressed genes in the fission yeast under nitrosative stress, cells were treated with two different doses of pure NO donor i.e. 0.1 mM and 1 mM for 15 min. It was previously reported that fission yeast *Schizosaccharomyces pombe* cells are much more sensitive to a concentration of 3 mM of detaNONOate than to 1 mM [1], [2] in terms of cell growth, lowering the mitotic index, while cell viability is maintained at 95%. The differentially expressed genes are listed in Table 1. Treatment of the wild type *S*. *pombe* cells with NO donor compound detaNONOate at a concentration of 1 mM resulted in 9 genes to be repressed while 30 genes were identified as induced (Table 2). The differential expressions of 8 genes were confirmed by Real-Time (RT) PCR analysis. The gene expression profiles (up regulation or down regulation) obtained by differential display analysis and by RT-PCR are similar as listed in Table 3. Gene Ontology (GO) terms that were enriched in the differentially expressed gene lists were searched using the online tool DAVID. In order to identify the different pathways affected under nitrosative stress, the information provided in KEGG was referred. Genes were classified as belonging to the different pathways that were affected upon treatment with pure NO donor compound. Fig. 1 shows the 18 pathways that were significantly affected (*p*<0.01) when the wild type *S*. *pombe* cells were treated with 0.1 mM concentration of pure NO donor detaNONOate for 15 min at 30 °C. Fig. 2 shows the 24 pathways that were significantly affected (*p*<0.01) when the cells were treated with 1 mM concentration of pure NO donor under similar conditions.

## 2 Experimental design, materials and methods

### 2.1 Chemicals

All components for the culture medium of *S*. *pombe* were purchased from Becton–Dickinson (USA). Regular laboratory reagents were purchased from the Sigma Chemical Company (USA) unless otherwise noted. Nitric oxide donor compound detaNONOate was purchased from the Cayman Chemical Company (USA).

### 2.2 Strains, media and treatment of the cells with NO donor compound

Wild type *S*. *pombe* (972*h*^*−*^) cells were grown in a YES medium (0.5% w/v Yeast extract, 3.0% w/v Dextrose with supplements as required) at 30 °C with shaking at 140 rpm. The NO donor compound detaNONOate (Z)-1-[N-(2-aminoethyl)-N-(2-ammonioethyl)amino]diazen-1-ium-1,2-diolate was prepared as a stock solution of 100 mM in 0.01 N NaOH. For the differential display experiments, the early exponential phase cells (O.D. at 600 nm=0.5) were harvested from the medium and re-suspended to double density in 1X Phosphate buffered saline (PBS; pH 7.4). The stress treatment was done in PBS to minimize the scavenging of NO by constituents of rich medium, such as yeast extract during the short time of exposure of the cells to NO. DetaNONOate was prepared as a stock solution of 100 mM in 0.01 N NaOH and added to the PBS (pH 7.4) at concentration of 0.2 mM and 2 mM. This solution was incubated at 30 °C, at 140 rpm, for 1 h, to ensure a stable release of NO into the solution prior to the addition of cells. The double-density cell suspension was added to the PBS solution containing detaNONOate, to a final density of 1×10^7^ cells/ml and a final concentration of 0.1 mM and 1 mM detaNONOate is thus achieved. Stress treatments were done for exactly 15 min at 30 °C in flasks shaking at 140 rpm. Controls were performed by adding cell suspension to a decomposed detaNONOate solution (final concentration 1 mM), to ensure that the effects upon treatment are due the nitric oxide that is released into the solution and not due to any of the by-products produced by degradation of detaNONOate.

### 2.3 RNA isolation protocol

The protocol for RNA isolation was followed from the Nurse Lab manual available in web (http://research.stowers.org/baumannlab/documents/Nurselab_fissionyeasthandbook.pdf). This method was used with minor modifications. The cells were harvested at 3000 rpm at 25 °C. Then the cells were washed with phosphate buffered saline (PBS; pH 7.4) once. The pellet was re-suspended in 100 µl cold RNA extraction buffer (50 mM Tris–HCl; pH 8.0, 100 mM EDTA pH 8.0, 200 mM NaCl), 100 µl cold phenol:chloroform (1:1) and 5 µl 10% SDS. The tubes were vortexed vigorously after adding acid washed glass beads for 5 min at 4 °C, another 200 µl RNA extraction buffer and 300 µl phenol:chloroform (1:1) were added and properly mixed by vortexing. The tubes were centrifuged at 13,000 rpm for 5 min at 4 °C and the upper aqueous phase was collected. The extraction step was repeated two times with phenol:chloroform (1:1) and finally to the aqueous phase collected, ammonium acetate was added to a final concentration of 2.5 M along with 2.5 volumes of ethanol. The tubes were kept at −20 °C for overnight for RNA precipitation. The precipitated RNA was washed with 70% ethanol twice and dissolved in RNase free water. RNA was checked by measuring O.D. at 260 nm and 280 nm and their ratio after 1:500 dilutions. The concentration of RNA was calculated taking 1 O.D. correspond to 40 µg of RNA/ml.

### 2.4 Treatment with RNase free DNaseI

Isolated RNA was subjected to DNaseI (Thermo) treatment to exclude the possibilities of any genomic DNA contamination. To an RNase free tube the following were added: Total RNA: 1 µg; 10X reaction buffer with MgCl_2_: 1 µl; DNase I (RNase free): 1 µl (1 unit); DEPC treated water: up to 10 µl. These tubes were incubated at 37 °C for 30 min. 1 µl of 50 mM EDTA was added to each of the tubes and further incubated at 65 °C for 10 min. The prepared RNA (concentration 0.1 µg/µl) was used as a template for Reverse Transcriptase PCR.

### 2.5 Reverse transcription of mRNA

Reverse transcription PCR was done using RNAimage kit of GenHunter Corporation (Nashville, TN, USA). Three reverse transcription PCR reactions were set for each RNA sample. Each PCR tube contained one of the three different one base anchored H-T_11_M primers (where M may be G, A, or C). The reactions were set according to the protocol: (For 20 μl final volume) DNase free water: 9.4 μl; 5X RT buffer: 4 μl; dNTP (250 μM): 1.6 μl; Total RNA (DNA free): 2.0 μl (0.1 μg/μl freshly diluted); H-T_11_M (2 μM): 2 μl. A core mix was prepared without a RNA template for each anchored oligo-dT primer. Thermal cycler (Applied Biosystems) was programmed as: 65 °C for 5 min >37 °C for 60 min >75 °C for 5 min >4 °C. After the tubes were at 37 °C for 10 min, thermal cycler was paused and 1 μl MMLV Reverse Transcriptase (Thermo) was added to each tube. Then the tubes were quickly mixed well by finger tipping and the reaction was continued. After the reaction, the tubes were stored in −20 °C.

### 2.6 Polymerase Chain Reactions using degenerate primers

The reactions were set according to the protocol (For 20 μl final volume for each primer pair combination): DNase free water: 10 μl; 10X PCR buffer: 2 μl; dNTP (25 μM): 1.6 μl; H-AP primer (2 μM): 2 μl; H-T_11_M (2 μM): 2 μl; RT-mix (It has to contain the same H-T_11_M used for PCR): 2 μl; α-[^33^P]dATP (2000 Ci/mmole): 0.2 μl; Taq DNA polymerase (Thermo) (5 u/μl): 0.2 μl. Core mix was prepared to avoid pipetting errors. Thermal cycler was programmed as: 94 °C for 30 s >40 °C for 2 min >72 °C for 30 s for 40 cycles followed by 72 °C for 5 min >4 °C. The PCR products thus obtained were radioactive and were properly stored at −20 °C.

### 2.7 6% denaturing polyacrylamide gel electrophoresis

6% denaturing (with 8 M urea) polyacrylamide gel was prepared in 1X Tris/Borate/EDTA buffer (TBE; pH 8.3) using Bio-Rad Laboratories׳ Sequencing grade gel apparatus. 3.5 μl of each sample was mixed with 2 μl of loading dye and incubated at 80 °C for 2 min. These samples were loaded onto the denaturing gel. Prior to loading the samples, each well of the gel was properly flushed with the 1X TBE buffer using a sterile syringe to remove the urea from the wells. The samples were electrophoresed for about 4 h at 60 W constant power (voltage not to exceed 1700 V) until the xylene dye (slower moving) reaches the bottom. After that the power was turned off and the gel was blotted on a piece of 3 M paper. The gel was covered with a plastic wrap, and dried under vacuum on a gel dryer (Hoefer Inc., USA) at 80 °C for 1 h. It is to be noted that since the DNA is acid labile, the gel should not be fixed in methanol/acetic acid otherwise it will cause difficulty in cDNA re-amplification.

The dried gel was exposed to x-ray film for 72 h; the cassette containing the dried gel and the film was placed inside a −80 °C freezer to minimize diffusion and distortion of the bands and then developed. The autoradiogram was analyzed for the presence of up regulated bands in the treated lanes compared to the control and the bands of interest were marked accordingly. Using a sharp and sterile surgical blade the necessary bands of interest were excised from the dried gel after correct orientation of the gel below and the autoradiogram above and the gel pieces were stored in numbered sterile micro-centrifuge tubes at −20 °C.

### 2.8 Extraction and re-amplification of DNA from the excised bands of interest

The DNA contained inside the gel pieces was extracted by adding 100 µl of sterile water to each of the tubes and allowing the gel pieces to stand for 10 min, the tubes were sealed with Parafilm M (USA) and boiled in a water bath for 15 min. A short spin was given to collect all condensation and the supernatant was transferred to newly labeled tubes into which 10 µl of 3 M Sodium acetate (NaOAc), 5 µl of Glycogen (10 mg/ml) and 450 µl of absolute ethanol were added to precipitate out the DNA from solution, for quick precipitation, the tubes were placed in a −80 °C freezer for 30 min. The tubes were centrifuged at 4 °C for 10 min at 10,000 rpm to collect the DNA pellet and the excess NaOAc was washed away by using 85% ethanol. Finally the DNA pellet was dissolved in 10 µl of sterile water and used for further re-amplification by PCR.

Each of the DNA bands isolated and precipitated out by the above mentioned protocol were re-amplified using the same set of primers. The PCR was done in a 40 µl reaction volume according to the protocol mentioned: DNase free water: 18 µl; 10X PCR buffer: 4 µl; MgCl_2_ (25 mM): 2.4 µl; dNTP mix (250 μM): 3.2 µl; H-AP primer (2 μM): 4 µl; H-T_11_-M primer (2 μM): 4 µl; DNA: 4 µl; Taq DNA polymerase (5 u/μl): 0.4 µl. Core mix was prepared to avoid pipetting errors. Thermal cycler was programmed as: 94 °C for 30 s >40 °C for 2 min >72 °C for 30 s for 40 cycles followed by 72 °C for 5 min >4 °C. The PCR products were run on a 1.5% agarose gel and checked for the presence of single bands.

### 2.9 Cloning the inserts into vector and transformation into competent cells

The PCR amplicons obtained were ligated into cloning vector pGEM-T easy (Promega). Since the multiple cloning site of this vector is located inside the coding region for the β-galactosidase gene, successfully cloned inserts will show defective translation for the β-galactosidase gene and hence those colonies will not be able to utilize X-gal as their substrate and hence produce white colonies onto IPTG/X-gal plates of Luria-Bertani Agar+Ampicillin which are selected and further grown in Luria-Bertani (LB) broth containing Ampicillin and the plasmids were isolated. The ligation protocol followed was as follows: 2X ligation buffer: 5 µl; pGEM T-easy vector (50 ng): 1 µl; DNA insert: 2.5 µl; T4 DNA ligase (3 u/µl): 1 µl. Total reaction volume was 10 µl. The tubes were mixed well and incubated at 4 °C overnight for increasing the number of recombinant colonies after transformation.

The protocol followed for transformation into competent cells is hereby stated. The strain of *Escherichia coli* chosen was XL1 Blue from Stratagene. The *lacIqZΔM*15 gene on the F′ episome allows blue–white screening for recombinant plasmids. The vials of competent cells (100 µl/tube) were taken out of −80 °C freezer and immediately set on ice. The ligated product was added to the competent cells and the tubes were set on ice for 4 h with occasional mixing by tapping gently. Heat shock was applied to the cells at 42 °C for exactly 45 s and immediately set back on ice. Fresh LB broth (without antibiotic) was added to each tube in a volume of 900 µl to make the total volume 1 ml and the tubes were incubated at 37 °C for 30 min with shaking at 140 rpm. Meanwhile LB agar plates containing Ampicillin at concentrations of 100 µg/ml were plated with 40 µl IPTG (0.1 M) and 40 µl X-gal (20 mg/ml) for blue/white screening of the transformed cells. After removing the transformed cell tubes from the incubator, those cells were plated onto IPTG/X-gal containing LB agar+Amp plates and incubated at 37 °C for 12 h.

### 2.10 Screening of white colonies for plasmid isolation

Since the multiple cloning site in the cloning vector was in the middle of the β-galactosidase gene, successfully ligated inserts would give defective transcription of the same thus the *E*. *coli* cells harboring such plasmids would not be able to utilize the substrate X-gal present on the plates and thus would remain white while the colonies metabolizing X-gal would turn blue. Our interest thus obviously remains in the white colonies for each of our inserts. A single white colony was inoculated into 2 ml of Luria-Bertani broth containing 0.1% Ampicillin and grown at 37 °C for 12 h under shaking conditions. The resulting culture was used for isolation of the plasmid containing our insert. The method used for plasmid isolation was followed as per instructions of Qiagen Mini spin Plasmid prep kit (Qiagen).

The isolated plasmids were measured spectrophotometrically at wavelengths 260 nm and 280 nm and the concentration determined by assuming that double stranded DNA with optical density 1 at 260 nm has a concentration of 50 μg/ml. Nucleic acids absorb maximally at 260 nm while proteins absorb maximally at 280 nm, the optical density ratios at 260 nm and 280 nm gives the indication of protein contamination in the plasmid preparation. The concentrations obtained for the plasmids were generally in the range of 250–300 ng/μl.

### 2.11 Checking for the presence of inserts by restriction digestion with EcoR1

Besides blue–white screening of transformed *E*. *coli* colonies, we checked for the presence of inserts in our isolated plasmids by digesting the plasmids with restriction endonuclease EcoRI (New England Biolabs). The protocol we followed for the restriction digestion was as follows: 10X NEB buffer: 3 µl; EcoRI (20,000 u/ml): 0.5 µl; Plasmid DNA: 1 µl; DNase free water: 25.5 µl. Total volume of the reaction was 30 µl. The tubes were incubated at 37 °C in a water bath for 1 h and the products were checked on a 1.5% agarose gel.

### 2.12 Sequencing of the inserts inside the plasmids isolated

The inserts cloned into pGEM-T easy vector were sequenced using M13 forward and reverse primers having sequences 5′GTAAAACGACGGCCAGTG3′ and 5′GGAAACAGCTATGACCATG3′ respectively. The reason for cloning our inserts into such a vector prior to sequencing is that this increases the read lengths of the sequences. In our case, differential display yields fragments of genes merely 150–180 bp long which we separate on a Urea-PAGE, excise and use this DNA for the other downstream applications. We cannot afford to lose any part of our insert׳s sequence due to primer binding or any other reading errors, as shortening of sequences further would give low stringency results upon matching with public databases. Thus using cloning vectors provides a better option for sequencing short insert lengths as in our case where the primer binds to the vector and covers the region containing the insert and a part of the vector too.

Sequencing PCR was performed using Applied Biosystems Big Dye Terminator cycle sequencing kit v3.1 on an Applied Biosystems 9700 thermal cycler using the protocol mentioned hereby: Big dye ready mix: 1 µl; 5X reaction buffer: 2 µl; M13 Primer (Fwd or Rev) (3.2 pmol): 1 µl; DNA template (plasmid): 100 ng; DNase free water upto 10 µl. Each sample was sequenced using both forward and reverse primers and sequences matched by database searches. The program followed for the PCR was as follows: 98 °C for 5 min >(96 °C for 10 s >50 °C for 5 s >60 °C for 4 min for 30 cycles) >4 °C.

### 2.13 Sequencing PCR product purification and sequencing of purified PCR product

i) Master Mix I: 10 μl MilliQ water+2 μl of 125 mM EDTA (pH 8) (added to each tube)
ii) Master Mix II: 2 μl 3 M NaOAc (pH 4.6)+50 μl 100% ethanol (added to each tube).

12 μl of Master Mix I was added to each of the PCR tubes containing sequencing product and the entire contents shifted to a 0.5 ml micro-centrifuge tube. 52 μl of Master Mix II was added to each of the tubes and vortexed mildly for 1 min after which the tubes were given a pulse spin. The tubes were incubated in the dark at room temperature for 15–20 min. The tubes were centrifuged at 13,000 rpm for 30 min at room temperature and the supernatant was discarded. 100 μl of 70% ethanol was added to the pellet and centrifuged at 13,000 rpm for 5 min at room temperature. The pellet was dried for 1 h in the dark at room temperature. The tubes containing the washed pellet were stored at −80 °C for sequencing.

On the day of sequencing, 20 μl of Hi-Dye Formamide from Applied Biosystems were added to each of the tubes and vortexed to dissolve the pellet completely, incubated in the dark for 15 min at room temperature. The tubes were placed on a dry bath at 95 °C for 2 min and immediately placed on ice to chill. The entire contents of each tube were loaded into sequencing plate and separated by capillary electrophoresis on an Applied Biosystems 3700 sequencer.

### 2.14 Preparation of the differentially expressed gene lists

The DNA sequence data obtained was analyzed using Chromas Lite software by retrieving the sequences in FASTA format and matching against NCBI database using the BLAST algorithm. During the database search only the highly similar sequences were considered. Since only the differentially regulated transcripts which were identified from the autoradiogram were excised and further processed, identification of the genes from database searches allowed us to build the list of the differentially expressed genes under nitrosative stress.

### 2.15 Validation of differential gene expression by Real Time PCR analysis

The cell growth, stress conditions and RNA extraction methods have been previously described. The first strand cDNA was synthesized using 400 ng of total RNA in a 20 μl reaction volume, using the Verso cDNA synthesis kit (Thermo). The primers for the genes (Table 4) whose expression was checked were added to a final concentration of 600 nM in a total reaction volume of 50 μl containing 25 μl of Power SYBR Green PCR master mix (Applied Biosystems). The reference housekeeping gene considered was *act*1 coding for Actin. The instrument used was the 7300 Real-Time PCR System (Applied Biosystems). All the results were generated from two independent biological repeats and for each biological experiment three technical repeats were performed. Relative quantification of gene expression was performed by the 2^−ΔΔCt^ method as described in a previous report [3]. The PCR conditions consisted of denaturation at 95 °C for 5 min, followed by 40 cycles of denaturation at 94 °C for 30 s, annealing at 60 °C for 30 s and extension at 72 °C for 30 s, and continued extension at 72 °C for 5 min.

### 2.16 Identification of the affected pathways from the differentially expressed genes

Online tool DAVID (http://david.abcc.ncifcrf.gov/) [4], [5] was used to find out the affected pathways among the differentially expressed gene lists. The gene lists were uploaded with selecting the background as all the genes of *S*. *pombe*. Functional Annotation Chart was visualized using the *p*-value threshold <0.01 and count 2. The information regarding the affected pathways was obtained from Kyoto Encyclopedia of Genes and Genomes (KEGG) within the analysis in DAVID, using the mentioned thresholds.

## Figures and Tables

**Table 1:** Differentially expressed genes upon 0.1 mM treatment with NO donor.

**Gene name**	**Systematic ID**
**Downregulated genes**	
*rad*17 checkpoint gene	SPAC14C4.13
Protein disulfide isomerase (predicted) (SPAC17H9.14c), mRNA	SPAC17H9.14c
60S ribosomal protein L36/L42 (*rpl*42), mRNA	SPAC15E1.03
Acyl-coA desaturase (predicted) (SPCC1281.06c), mRNA	SPCC1281.06c
Mitochondrial ribosomal protein subunit L13	SPBC16G5.04
Alpha-glucosidase Agl1	SPAPB24D3.10c
Mdm10/Mdm12/Mmm1 complex subunit Mdm10 (predicted) (*mdm*10), mRNA	SPAC17H9.17c
60S ribosomal protein L28/L44 (predicted) (*rpl*44), mRNA	SPAC1687.06c
*ade*10 gene	SPCPB16A4.03c
**Upregulated genes**	
ATP-dependent DNA helicase (predicted) (SPBC23E6.02), mRNA	SPBC23E6.02
Hsk1-Dfp1 kinase complex regulatory subunit Dfp1 (*dfp*1), mRNA	SPCC550.13
*csx*2+gene, partial cds	SPBC17G9.08c
Alpha-glucosidase *gto*1	SPAC1039.11c
SUMO E1-like activator enzyme Fub2	SPBC16H5.03c
6-phosphofructokinase (predicted)	SPBC16H5.02
Ribosome biogenesis protein Brx1 (*brx*1), mRNA	SPBC800.06
60S ribosomal protein L23	SPAC3G9.03
3-oxoacyl-[acyl-carrier-protein] reductase Oar2 (predicted)	SPAC3G9.02
Ran GTPase binding protein Mog1	SPCC1840.01c
1,3-beta-glucan synthase subunit Bgs4	SPCC1840.02c
rRNA processing protein Rrp7 (predicted)	SPBC776.17
Transcription factor TFIIH complex subunit Pmh1	SPBC776.18c
*rec*12 gene, complete cds	SPAC17A5.11
Protein phosphatase regulatory subunit Pab1	SPAC227.07c
Malate dehydrogenase (SPCC306.08c), mRNA	SPCC306.08c
Methionyl-tRNA formyltransferase Fmt1 (predicted) (*fmt*1), mRNA	SPAC1805.09c
Conserved fungal protein (*rds*1), mRNA	SPAC343.12
WTF element Wtf20	SPCC1906.04
ZF-CCCH type zinc finger	SPCC1739.01
19S proteasome regulatory subunit Rpn3 (*rpn*3), mRNA	SPBC119.01
DUF1761 family protein (SPAC15E1.02c), mRNA	SPAC15E1.02c
*rec*8 gene, complete cds	SPBC29A10.14
Nucleoporin Nup146 (*nup*146), mRNA	SPAC23D3.06c
Mitochondrial FAD transporter (predicted)	SPBC27B12.09c
Mitochondrial TOM complex subunit Tom7	SPBC27B12.10c
Single-stranded telomeric binding protein Tcg1	SPBC660.11
Mannosyl-oligosaccharide 1,2-alpha-mannosidase (*mns*1), mRNA	SPAC2E1P5.01c

**Table 2:** Differentially expressed genes upon 1 mM treatment with NO donor.

**Gene name**	**Systematic ID**
**Downregulated genes**	
*rad*17 checkpoint gene	SPAC14C4.13
Protein disulfide isomerase (predicted) (SPAC17H9.14c), mRNA	SPAC17H9.14c
60S ribosomal protein L36/L42 (*rpl*42), mRNA	SPAC15E1.03
Acyl-coA desaturase (predicted) (SPCC1281.06c), mRNA	SPCC1281.06c
Mitochondrial ribosomal protein subunit L13	SPBC16G5.04
Alpha-glucosidase Agl1	SPAPB24D3.10c
Mdm10/Mdm12/Mmm1 complex subunit Mdm10 (predicted) (*mdm*10), mRNA	SPAC17H9.17c
60S ribosomal protein L28/L44 (predicted) (*rpl*44), mRNA	SPAC1687.06c
*ade*10 gene	SPCPB16A4.03c
**Upregulated genes**	
Squalene synthase Erg9 (*erg*9), mRNA	SPBC646.05c
Protein disulfide isomerase (predicted) (SPAC17H9.14c), mRNA	SPAC17H9.14c
Cell wall protein Asl1, O-glucosyl hydrolase (predicted) (*asl*1), mRNA	SPAC13G6.10c
Poly(A) polymerase Cid14 (*cid*14), mRNA	SPAC12G12.13c
Actin cortical patch component, with EF hand and WH2 motif	SPAC25G10.09c
Tropomyosin	SPAC27F1.02c
Ribosome biogenesis protein (predicted) (SPCC550.15c), mRNA	SPCC550.15c
SUMO-targeted ubiquitin-protein ligase E3 Slx8 (predicted) (*slx*8), mRNA	SPBC3D6.11c
Nuclear pore associated protein Thp1-Sac3 complex subunit (predicted)	SPBC1105.07c
Phosphomannomutase Pmm1 (*pmm*1), mRNA	SPAC1556.07
Multi-copy suppressor of Chk1 (*msc*1), mRNA	SPAC343.11c
DNA polymerase alpha catalytic subunit (*pol*1), mRNA	SPAC3H5.06c
60S ribosomal protein L11	SPAC26A3.07c
60S ribosomal protein L30	SPAC9G1.03c
Ribosome biogenesis protein Sqt1	SPAC25H1.08c
Heat shock protein Pdr13	SPAC57A7.12
C-22 sterol desaturase Erg5	SPAC19A8.04
Serine hydrolase	SPAC22A12.06c
Clr6 histone deacetylase complex subunit Pst2	SPAC23C11.15
Polo kinase Plo1	SPAC23C11.16
Septin Spn4	SPAC9G1.11c
Phosphoric monoester hydrolase	SPAC823.14
Rho-type GTPase activating protein Rga5	SPBC17F3.01c
Vacuolar carboxypeptidase Y *cpy*1	SPAC19G12.10c
N-acetylglucosaminyldiphosphodolichol N-acetylglucosaminyltransferase Alg13 (predicted)	SPAC56E4.02c
Aromatic aminotransferase	SPCC569.07
DNA replication factor C complex subunit Rfc3	SPAC27E2.10c
ATP-dependent RNA helicase Prp43 (predicted)	SPBC16H5.10c
MBF transcription factor complex subunit Rep1	SPBC2D10.06
Thioredoxin reductase *trr*1	SPBC3F6.03

**Table 3:** Validation of gene expression obtained from differential display by Real Time PCR.

**Treatment with 0.1 mM detaNONOate**		
**Gene name**	**Regulation in differential display**	**Regulation and fold change by RT-PCR**
*dfp*1	Up	Up 1.9 fold
*fub*2	Up	Up 2.3 fold
*rec*12	Up	Up 1.7 fold
*rec*8	Up	Up 1.9 fold

**Treatment with 1 mM detaNONOate**		
**Gene name**	**Regulation in differential display**	**Regulation and fold change by RT-PCR**
*trr*1	Up	Up 2.4 fold
*rfc*3	Up	Up 1.5 fold
*rga*5	Up	Up 2.2 fold
*pdr*13	Up	Up 1.8 fold

**Table 4:** Primer sequences of the genes validated by Real-Time PCR.

**Gene name**	**Forward primer (5′---->3′)**	**Reverse primer (5′---->3′)**
*dfp*1	GGTCTATGGAAAAGCTTTGCA	GATAAATCGCGTTCGTTCGTT
*fub*2	ACCGAAGGATTACCTTACCAA	TAACGATTCTTTCCGACGAAG
*rec*12	CTTAACGTTGAAGCATCTGCT	ACCAAGACCCATTTTGCTGT
*rec*8	GGACCATTTCACTTCAAAACC	ACATGTAGATCCACAGAAGGT
*trr*1	TTTTGGCTACTGGTGCTTCC	AACGACAGCAAGAGGCTTGT
*rfc*3	TTTGAAGCACTAGACGAGCTT	CACCAGTTTTAATGCTCGCTA
*rga*5	TACAGACAATGAGCGAGTGGA	AGCAGTCATCAGGTTCTTCGA
*pdr*13	GCGTGTACTTAGCAATGGTAC	ACAGCTTCGGTTACAAGATCG
*act*1	GTTATGTCTGGTGGTACCACT	GATCCACCAATCCAGACAGA
